# Authors’ Response to Peer Reviews of “Satisfaction With Health Care Services at the Pediatric Specialist Clinic of the National Referral Center in Malaysia: Cross-sectional Study of Caregivers’ Perspectives”

**DOI:** 10.2196/37117

**Published:** 2022-05-25

**Authors:** Thinakaran M Selvarajah, Eiko Yamamoto, Yu Mon Saw, Tetsuyoshi Kariya, Nobuyuki Hamajima

**Affiliations:** 1 Department of Healthcare Administration Nagoya University Graduate School of Medicine Nagoya University Nagoya Japan; 2 Hospital Putrajaya Ministry of Health Putrajaya Malaysia; 3 Nagoya University Asian Satellite Campuses Institute Nagoya Japan

**Keywords:** pediatrics, caregivers, health care services, public hospital, Malaysia, public-private-partnership, children

*This is the authors’ response to peer-review reports for "Satisfaction With Health Care Services at the Pediatric Specialist Clinic of the National Referral Center in Malaysia: Cross-sectional Study of Caregivers’ Perspectives."*

## Reviewer Anonymous [1]

### Round 1 Review

#### General Comments

Thanks for the opportunity to review this manuscript [2] entitled “Caregivers’ Perspective—Satisfaction With Healthcare Services at the Paediatric Specialist Clinic of the National Referral Centre in Malaysia.” The authors report on an important topic, and their research work will contribute to the existing literature. Overall, the manuscript is well written with enough details in different sections. The tables are informative. The following are comments/concerns for the authors to consider.

#### Specific Comments

1. Abstract: include data/numbers in the Results section rather than general summary statements

Response: Amendment done with relevant data/numbers

2. Introduction: include any a priori hypotheses

3. Introduction: to support the rationale for the review, the authors should include additional recent promising evidence that supports the feasibility, acceptability, and efficacy of digital health interventions in different chronic medical conditions to provide context for the applicability of lessons learned in the study across other fields [3-8].

Response: The sample articles provided focus on the use of mobile health (mHealth)/digital health/technology/telemedicine, whereas this paper is on caregiver satisfaction by simply using the SERVQUAL questionnaire.

“Many studies conducted at public health care facilities in Malaysia have shown a high level of patient satisfaction with the services provided (19). However, to our best knowledge, no studies have been conducted on caregivers’ satisfaction in MoH pediatric outpatient clinics or facilities. This study, therefore, aims to ascertain the prevalence and factors influencing satisfaction and to identify areas of dissatisfaction among caregivers at the Paediatric Specialist Clinic of Tunku Azizah Hospital.”

4. Discussion: two recent reviews focused on pediatric/adolescent care and COVID-19 with mHealth/eHealth and adolescent/children psychosocial well-being, both worth discussing [9,10]

5. Discussion: the authors could consider including a paragraph on study strengths.

6. Discussion: it is critical to discuss the value of including direct patient input in the development of mHealth interventions, and other key considerations for end users should be sought early on in the process of app or digital health intervention design to ensure long- and short-term engagement [11-14].

Response: The instances given here are speaking from an angle of mHealth, which does not correlate with our paper.

7. Discussion: the authors should expand and elaborate more on how their findings support or contrast available literature and provide suggestions for future research directions that would address existing knowledge gaps.

8. Discussion: the authors should also acknowledge the lack of economic data to support the use of digital health interventions to date [15,16].

Response: Mentioned at the end of the Discussion section:

“Routine satisfaction assessments should be conducted using improvised questionnaires or other tried-and-true methods to identify unsatisfactory domains that require substantial improvements. These measures will ensure that the services provided are in line with the Ministry of Health’s mission of providing quality integrated, people-centered health care to the masses. Future studies may be able to compare additional hospitals that use the PFI model, as well as provide more information about the variations discovered in this study.”

### Round 2 Review

No additional comments.

## Reviewer BX [17]

### Round 1 Review

#### General Comments

This paper describes interesting research about factors affecting the satisfaction of caregivers at a national referral center. I really liked the research performed and the article. Nevertheless, I think that there are some minor aspects that perhaps could be better described so the readers can better understand the results and their external validity. The authors do explain the limitations adequately, but perhaps some aspects could be clarified within the main text of the article.

#### Specific Comments

##### Major Comments

1. In Methods, the authors write that “This cross-sectional study was conducted at the Tunku Azizah Hospital, Kuala Lumpur, Malaysia. Subjects were caregivers to children seen with an appointment at the clinic.” They also write that “This study was conducted at the hospital’s Paediatric Specialist Clinic by convenience sampling. Self-administered, structured questionnaires were distributed to consenting participants. Subjects who agreed to participate were given questionnaires after seeing the doctor and while waiting for the date of their next consultation.”

Selection bias is probably the most important limitation of this research. Selection bias is almost unavoidable, so the authors must make a considerable effort to clearly describe where they obtain the sample from, so the readers can have a clear idea of the main features of that sample, which also should be described. To better understand the results (and therefore the conclusions), it would be very interesting to know, in more detail, how the patients were chosen, the attrition rate, or other factors related to the sample selection. Therefore, I would propose that the authors better describe where the sample is obtained from and how they were chosen.

Response: Mentioned in the Data Collection section:

“This study was conducted at the hospital’s Paediatric Specialist Clinic by convenience sampling using a self-administered structured questionnaire. Every third registering caregiver was identified and given the questionnaires after seeing the doctor and while waiting for the date of their next consultation. Upon completing the questionnaire, participants were instructed to put it into an enclosed envelope. The sealed envelope is then passed to the nurse at the clinic counter.”

2. In that same section, the authors write that “A total of 600 questionnaires distributed to the clinic, and we received 502 responses, giving a rate of 83.7%. Of these 502 responses, 43 were unusable and were excluded from this study, and the remaining 459 (91.4%) questionnaires were analysed. Some 2,238 patients were registered for an appointment at the clinic during this data collection period.”

It would be interesting if they describe in the article if they performed any sample size estimation and which method did they employ, in that case.

Response: Mentioned in the Methods (Participants) section:

“The minimum sample size required is 364, which was calculated using the Raosoft (2004) online sample size calculator with a 95% confidence level, 0.5 SD, margin of error (CI) of 5%, and population size of 6714 (the monthly patient average).”

3. The authors write that “This was part of a hospital-level survey assessing satisfaction among caregivers attending the clinic using the SERVQUAL instrument.”

They properly describe the dimensions of the questionnaire, but perhaps it would be useful to know if this tool has been validated (or has required transcultural adaptation) to be used with this specific sample.

Response: Mentioned in the Data Collection section:

“The analysis of gaps is based on the difference between service quality expectations and perception. It was modified, translated, and validated in line with the Malaysian health care setting (22).”

4. Despite these aspects, which are easily solvable, I think that this is a very interesting article that can be useful for other researchers.

##### Minor Comments

Some sentences and some paragraphs are perhaps a bit too long, and therefore, they are a bit confusing to read, but overall, the article is very well written.

## Abbreviations

mHealth: mobile health
